# Effects of inclined treadmill training on functional and cardiovascular parameters of stroke patients: study protocol for a randomized controlled trial

**DOI:** 10.1186/s13063-019-3298-3

**Published:** 2019-05-02

**Authors:** Raiff Simplício da Silva, Stephano Tomaz da Silva, Jesimiel Missias de Souza, Marianna Celeste Cordeiro de Figueiredo, Thaís Almeida Silveira Mendes, Maria Clara de Sena Nunes, Samara Katiane Rolim de Oliveira, Daiane Carla Rodrigues Cardoso, Raiza Gabriella da Câmara Silva, Débora Carvalho de Oliveira, Tatiana Souza Ribeiro

**Affiliations:** Laboratory of Intervention and Movement Analysis, Department of Physical Therapy, Federal University of Rio Grande do Norte, Avenida Senador Salgado Filho, 3000, Natal, Rio Grande do Norte 59078-970 Brazil

**Keywords:** Rehabilitation, Gait disorders, Exercise, Physical therapy modalities

## Abstract

**Background:**

Treadmill training has been widely used for gait recovery after stroke. Gait re-establishment is one of the main objectives of rehabilitation programs after stroke, aiming to acquire more functional patterns and increase walking speed, along with improvement in cardiovascular function. The aim of this study is to evaluate the effects of a treadmill gait training protocol on functional and cardiovascular variables in patients with chronic stroke.

**Methods:**

A single-blind randomized clinical trial will be conducted. The sample will consist of 36 patients, who will be allocated in three groups: control group (n = 12), experimental group 1 (n = 12), and experimental group 2 (n = 12). The intervention will occur for 6 consecutive weeks, three times a week, 30 min each session, in all groups. The control group will perform a treadmill gait training without inclination, experimental group 1 will perform a treadmill gait training with anterior inclination of 5%, and experimental group 2 will perform a treadmill gait training with anterior inclination of 10%. All participants will be assessed for sample characterization measures, gait speed, functional capacity, systemic arterial blood pressure, heart rate, peripheral oxygen saturation, exercise capacity, neuromuscular torque, and quality of life. Evaluations of outcome measures will occur at the end of the interventions (post-training) and after 1 month and 1 year after the end of the interventions (short- and long-term follow-up). Statistical analysis will be performed descriptively and inferentially. Alpha equals 5% will be considered for inferential analysis. Mixed analysis of variance with repeated measures will be used to compare outcome measures between groups and between baseline, post-training, and follow-up. Normality test (Shapiro–Wilk) and subsequently *t* test (or Mann–Whitney) will be used to compare groups during the same training session.

**Discussion:**

It is believed that treadmill training, especially treadmill training with anterior inclination, may result in improved exercise capacity in patients with stroke, reduced blood pressure and heart rate values, and an improvement in functional parameters with increased gait speed, functional capacity, quadriceps muscle torque, and quality of life.

**Trial registration:**

Registration in Brazilian Registry of Clinical Trials (ReBEC) identifier RBR-5ffbxz, date of registration October 25 2017.

**Electronic supplementary material:**

The online version of this article (10.1186/s13063-019-3298-3) contains supplementary material, which is available to authorized users.

## Background

Stroke is an important cause of death and incapacity around the world, affecting about 795,000 individuals per year overall [1]. It is estimated that 75% of all deaths caused by stroke occur in developing countries, and 80% of individuals with disability after stroke are in these countries [2]. In Brazil, cerebrovascular diseases resulted in about 100,000 deaths in 2014 [3], and data indicate that about 568,000 affected individuals remain with severe disability [4].

People affected by stroke commonly present motor or sensory changes (or both), unilateral or bilateral, loss of strength and coordination, visual and cognitive impairments, and perceptual and language deficits [5]. These changes promote difficulties in executing functional activities and reduced performance of daily living activities and a consequent reduction in social participation which can aggravate the presented clinical situation [6].

An important factor leading to functional disability after stroke consists of gait deficits [7]. Hemiparetic gait is characterized by slow, laborious, and uncoordinated movement of limbs, although different gait patterns can be noticed according to the degree of motor recovery [8]. Since gait coordination is affected, the ability to change the walking pattern in response to environmental demands is also affected [9]. Therefore, other daily locomotion activities such as going up and down stairs, walking on sloped surfaces, and making turns and changing direction to return to a specific place or to avoid or go around obstacles are impaired as well.

Stroke patients who present hemiparetic gait have shown low levels of physical activity [10]. Physical inactivity leads to a decline in aerobic capacity, a decrease in exercise tolerance, and a limitation in daily activities [11]. Therefore, there is a vicious cycle involving physical inactivity and functional decline [12]. It is important to emphasize that increases in heart rate (HR) and systemic arterial blood pressure (BP) occur after stroke due to high sympathetic nervous system activity [13], and an increase is also associated with a decrease in physical fitness. In this way, gait recovery is one of the main objectives of stroke rehabilitation programs, which aim to acquire more functional patterns, increase walking speed, and improve cardiovascular function [14–16].

Studies have indicated that treadmill training improves gait speed [12, 17] and cardiovascular parameters, decreasing the BP and HR of these patients [13, 18], and it is more effective than conventional rehabilitation in improving aerobic capacity after stroke. In addition, treadmill training has been shown to be effective in improving postural balance, functional mobility, and lower limb function in patients with stroke [19–22].

Treadmill training can be carried out according to several protocols and also associated with difficult factors such as anterior or posterior inclination of the treadmill, direction changes in movement (backward), and an increase in load in order to increase weight [23–25]. Specifically, regarding the treadmill anterior inclination, some studies have been carried out in patients with stroke [20, 23, 26–28] given that walking on inclined surfaces is an activity commonly faced by community-dwelling people with locomotor impairments [26]. All of these studies observed improvement in gait variables—such as increased walking speed and range of motion of lower limbs, longer paretic step length, and improved gait balance and symmetry—during or after training on an inclined treadmill. Authors point out that slope seems to promote beneficial changes in hemiparetic gait pattern, which can lead to a better adaptation of ambulant stroke subjects to real environments [26, 27]. In addition to gait parameters, Globas et al. [20] investigated cardiovascular parameters after a fitness training (3 months), verifying better cardiovascular fitness with training on inclined treadmill compared with conventional physiotherapy. However, the degree of treadmill anterior inclination as a resistance grading in a fitness training (≥6 weeks) has not been investigated.

Thus, there is a strong need to design, implement, and evaluate training protocols which include functional activities—such as gait, walking up or down stairs, and ramps—to improve the cardiovascular condition of individuals with neurological pathologies, particularly stroke. Since treadmill training may influence functional and especially cardiovascular outcomes of individuals with a prior health condition, it is important to consider these variables in evaluating post-stroke training protocol effects. Also, Mehrholz *et al*. [29], in a recent systematic review, suggest further studies including different intensities (such as inclination) for treadmill training after stroke, as improved walking speed and endurance had no long-term benefits. In this context, the following questions arise: (1) Does inclined treadmill training promote cardiovascular and functional parameters in individuals with chronic stroke, and is it effective and safe for these patients when compared with treadmill training without inclination? (2) If inclined treadmill training promotes these parameters, what level of treadmill inclination is the most effective for improving cardiovascular and functional variables of individuals with chronic stroke?

The proposal of the study is to investigate whether treadmill inclination influences functional and cardiovascular parameters compared with treadmill gait without inclination and whether there are differences in effects on cardiovascular or functional parameters (or both) according to the degree of treadmill inclination during gait training.

## Methods/design

### Study setting and subjects

The present study is a single-blind randomized controlled clinical trial that follows the recommendations of the Consolidated Standards of Reporting Trials (CONSORT) [30].

Data collection procedures will be performed at the Laboratory of Intervention and Movement Analysis (LIAM), Department of Physical Therapy, Federal University of Rio Grande do Norte (UFRN), in the city of Natal, Rio Grande do Norte, Brazil.

The study population will consist of patients with stroke diagnosis who live in the city of Natal or nearby. Volunteer selection will be carried out in stroke patient care places, which may be public referral centers or private institutions of the city. The selection can also be carried out via spontaneous demand by the voluntary search of stroke patients after project advertisement on social media.

### Sample size

Sample size was calculated by using an online calculator [31], and the variable gait speed (in meters per second) was adopted as the primary outcome measure. According to a previous study involving individuals with post-stroke hemiparesis who performed treadmill training with partial weight support and anterior inclination of 10° (experimental) and no inclination (control) [26], standard deviations observed in the experimental and control groups were 0.1 and 0.2, respectively. The sample size was calculated from these data to detect a difference in gait speed between the groups of 0.18 m/s (α = 5% and power = 80%) since the minimum detectable difference in the gait speed for patients with chronic stroke was 0.18 m/s [32]. A ratio between the control and experimental groups of 1:2 was considered, as it was verified that the smallest numbers needed to detect the expected difference would be 8 individuals in the control group and 16 individuals in the experimental group, totaling 24 participants. Given a dropout rate of about 40%, a sample total of 36 participants (12 in the control group and 24 in the experimental group) was determined.

### Inclusion and exclusion criteria

The participants will be selected according to the following criteria: (1) first episode of unilateral stroke (ischemic or hemorrhagic) resulting in walking deficits, (2) injury time equal to or greater than 6 months, (3) age between 20 and 70 years old, (4) ability to walk without personal assistance indoors (Functional Ambulation Category (FAC) scores equal to or greater than 3) [33], (5) walking speed on the ground equal to or less than 0.9 m/s (limited community walking), according to the categorization proposed by Fulk *et al*. [34] (2017), and (6) ability to understand and obey simple motor commands.

Exclusion criteria will include (1) pregnancy; (2) show instability in cardiac conditions (uncontrolled heart disease) or heart failure (New York Heart Association (NYHA) scores of at least 3 (or both) [35]; (3) presenting other clinical conditions affecting gait; (4) presenting pain or severe discomfort which hinders performing the proposed activities (or both); (5) presenting decompensation of systemic arterial pressure, systolic and diastolic values [36]; and (6) HR above the submaximal values allowed during training, maintained even after pauses, calculated by means of the formula [HRsub = 0.75 × (220 − age)] [37], where HRsub = submaximal HR.

### Randomization and blinding

Randomization sequence will be generated by computer [38], and three groups with blocks of 12 participants will be considered. This stage will be conducted by a researcher not involved in the study. This researcher will keep the randomization list confidential until the end of the study and will organize the allocation in sequentially numbered opaque envelopes. These envelopes will be sealed, and the randomization sequence will be according to the coding that will be created for study groups (control, experimental 1, and experimental 2).

The content of each envelope will be revealed only at the beginning of the training of each patient by study therapists in order to maintain allocation confidentiality. The same therapists involved in control group training will perform training in the experimental groups. Sample characterization measures and outcome measures will be evaluated by masked researchers in relation to group allocation. These researchers will be the same as those who enrolled participants in the study.

**Fig. 1:**
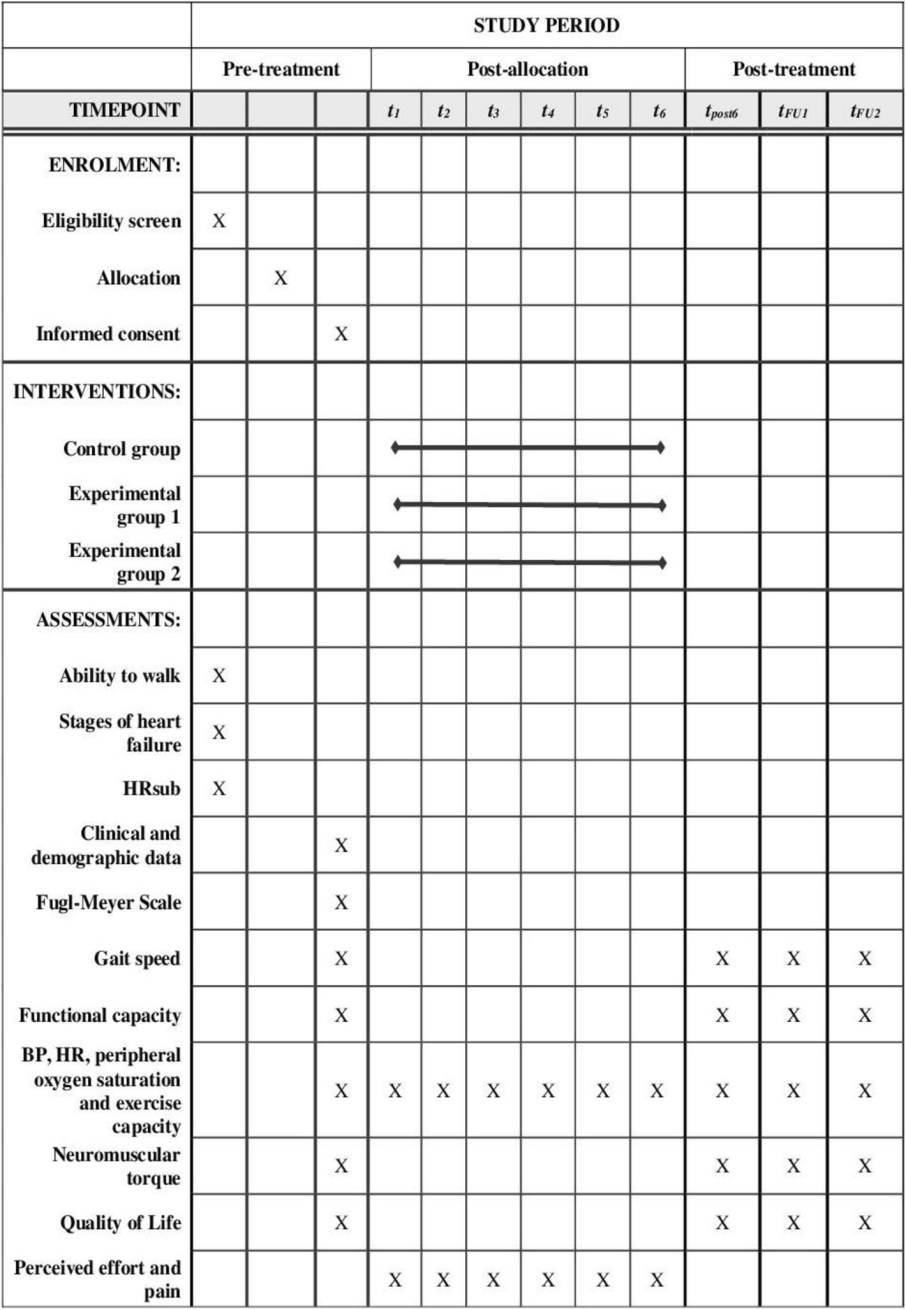
Schedule of enrollment, interventions, and assessments. *Abbreviations*: *BP* systemic arterial blood pressure, *HR* heart rate, *HRsub* submaximal heart rate, *t1* 1st week, *t2* 2nd week, *t3* 3rd week, *t4* 4th week, *t5* 5th week, *t6* 6th week, *t1month* short-term follow-up, *t12months* long-term follow-up, *tpost6* post-training

Only variables that will be collected during the training will be evaluated by study therapists (non-blind). Statistical analysis will be performed by one researcher, who will be blind to the allocation of patients in groups. Data will be tabulated with a predetermined encoding by another blinded evaluator, who will also check data (range check for data values). The present protocol has been prepared in accordance with relevant items from the SPIRIT (Standard Protocol Items: Recommendations for Interventional Trials) checklist (Additional file 1) and the SPIRIT figure (Fig. 1).

## Measures

### Sample characterization measures

- Clinical and demographic data: These will be evaluated by filling out a structured identification form about data such as paretic side, stroke type, age, gender, injury time, and drug treatments (including beta-blockers).
- Ability to walk: This will be evaluated by FAC, which consists of a sensitive and reliable instrument for gait evaluation in stroke patients with hemiparesis [33]. FAC ranks six levels of walking ability according to the amount of physical support required for this task. On this scale, the score can vary from 0 (unable to walk or needs the help of two therapists) to 5 (independent in locomotion).
- Motor function: It will be evaluated by Fugl–Meyer Scale, which allows us to analyze and quantify sensory-motor function after stroke [39]. Only motor function requirements will be used in this study, and it includes measuring movement, coordination, and reflex activity of the shoulder, elbow, wrist, hand, hip, knee, and ankle. Scores below 50 points indicate severe motor impairment; 50–84, outstanding; 85–95, moderate; and 96–99, mild [40].

### Outcome measures

Primary outcome measures considered for this study are the following:

- Gait speed: This will be measured by the 10-meter walking test [41]. This test consists of a simple measure which presents good reliability and reproducibility and is valid to evaluate physical mobility in a clinical or home environment [42].
- Functional capacity: This will be measured by the 2-min walk test [43]. This test has demonstrated adequate inter- and intra-examiner reliability in patients with stroke and has an advantage compared with the 6- and 12-min walk tests because it presents a time considered efficient and reduces fatigue effects in these patients [44].
- Cardiovascular parameters: BP will be checked by using a calibrated digital arm sphygmomanometer (Visomat Comfort III^®^, Incoterm, São Paulo, Brazil) on the non-paretic arm. HR will be measured by means of an HR meter (chest belt) (Polar Care^®^, Polar, São Paulo, Brazil). Derived variables such as mean BP, reserve HR, and predicted maximum HR will be calculated from BP and HR measured values. Physiological measures such as BP and HR are non-invasive parameters easily verified by using available devices in clinical practice. Exercise HR has been used as a physical fitness measure after stroke [45], and both HR and BP have been indicated to evaluate the cardiovascular response to exercise in individuals with stroke [14]. Also, peripheral oxygen saturation will be checked by using a pulse oximeter (Palpus 1 SA210, Rossmax, Taipei, Taiwan). Exercise capacity (energy expenditure) in metabolic equivalents (METs) will be estimated from HR by using the HR index (HR_INDEX_) in accordance with the equation developed by Wicks *et al*. [46] for patients with and without heart disease (including those taking beta-blockers).

Secondary outcome measures considered for this study are the following:

- Neuromuscular torque: Quadriceps muscle isometric torque peak of both lower limbs will be measured by using an isokinetic dynamometer (Biodex Multi-Joint System 3 pro^®^, Biodex Medical Systems, Shirley, NY, USA). The isokinetic dynamometer is considered a gold standard in muscular strength evaluation and is used to evaluate isometric torque peak and force in patients with stroke [47, 48]. Torque peak will be obtained with patients performing five isometric contractions of knee extension, interspersed with 30 s of rest, counting only the contraction which obtains the highest torque value. Each contraction should last 5 s, and the first and last seconds are not counted in the measurement.
- Quality of life: The assessment of quality-of-life perception will be performed through a quality-of-life assessment scale in stroke (Stroke-Specific Quality of Life Scale, or SS-QoL) [49]. This scale has 49 items distributed in 12 domains (energy, family role, language, mobility, mood, personality, self-care, social role, reasoning, upper limb function, vision, and work/productivity). It is valid and reliable in assessing quality of life after stroke, including in the Brazilian population [50].

### Participant monitoring measures

Participants will be monitored during interventions by not only cardiovascular parameter variables but also the following variable:

- Perceived effort and pain: Quantification of perceived effort and pain will be used as indicators to monitor exercise tolerance. They will be verified through the CR-10 (Category-Ratio Scale) Borg Scale [51], modified by Foster *et al*. [52] (2001).

## Evaluation procedures

The researchers will be trained before data collection procedures to ensure the reliability of measurements. It is important to highlight that these researchers will remain blind to group allocation throughout the study period.

Previously contacted patients with stroke or those who spontaneously search for the study will be evaluated according to inclusion criteria at our laboratory, where they will be informed about the research objectives and procedures to be carried out. Once they agree to participate in the study, they will be asked to sign a consent form.

The participants will first be submitted to a general approach to obtain clinical, demographic, and anthropometric data, and other measures of sample characterization will also be collected. Furthermore, the following outcome variables will be evaluated: gait speed, functional capacity, and muscle torque. Participants will subsequently remain at rest, sitting for at least 15 min. During this period, quality of life will be evaluated. Lastly, cardiovascular parameters will be obtained (at rest).

Outcome measures will be collected again at the end of the interventions (post-training), after 30 days of the end of the interventions (short-term follow-up), and after 1 year of the end of the interventions (long-term follow-up). Adverse events will be evaluated at follow-up.

## Experimental protocol

Participants will undergo all procedures described above on the day of initial evaluation and then the first intervention session will begin. Patients will be allocated according to groups in which they were randomly assigned. The group will be revealed when the envelope is opened by therapists.

## Study groups

Control group – treadmill gait training

**Fig. 2:**
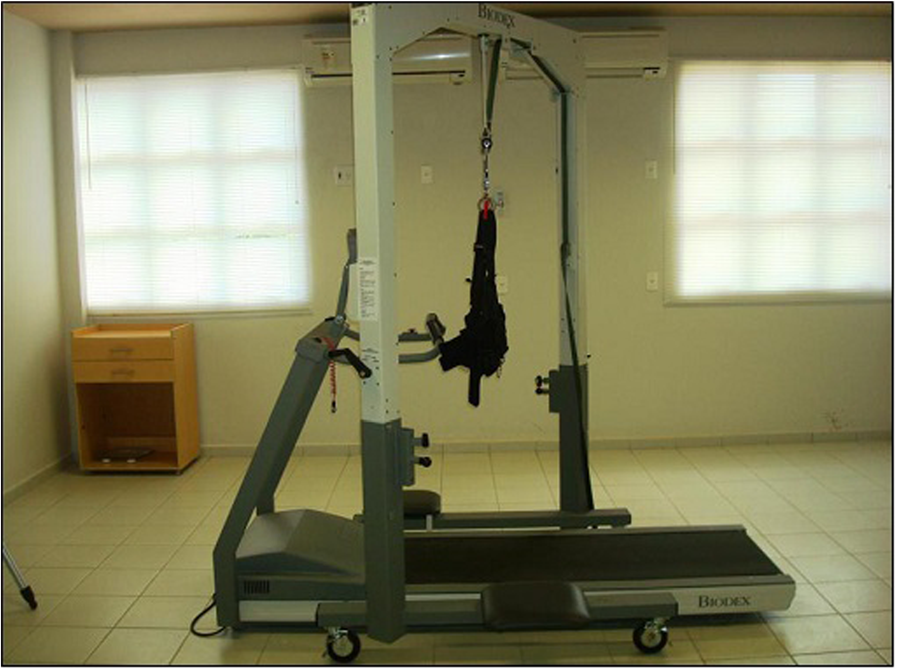
System for gait training: Gait Trainer System 2

The Gait Trainer System 2 (Biodex Medical Systems), which contains an electric treadmill with a walking area of 160 × 51 cm and an anterior bar, will be used for treadmill gait training (Fig. 2). This equipment allows one to change the speed with a minimum speed of 0.04 m/s and a maximum speed of 4.7 m/s, providing real-time data on speed and traveled distance. Associated with the treadmill is the partial weight support system (Unweighing System) consisting of a vest coupled to a body weight suspension mechanism. However, the vest will be used only for the purpose of providing safety and balance but will not provide any support [25].

The training protocol will follow what was used in a previous study, involving patients with subacute stroke, since the protocol used was safe and capable of increasing gait speed and distance covered on a treadmill [53]. In the first training session, participants will undergo a few minutes of treadmill training for familiarization and will be instructed to hold onto the anterior treadmill bar with their non-paretic hand to provide stability. The treadmill speed will be selected as the “maximum comfort”, meaning the maximum tolerated by the individual, while adequate posture is maintained throughout gait cycle without any compensation or muscle fatigue.

From the second session, participants will be encouraged to withdraw support from their non-paretic hand over the treadmill bar. In the following session, participants will be encouraged to increase the treadmill speed according to individual tolerance. Participants will be encouraged to achieve maximum speed at the beginning of the session. After reaching that speed, it will remain constant throughout the session. This procedure will be repeated at the beginning of all subsequent sessions.

The treadmill training sessions will last 30 min, and two small rest periods (about 3 min) will be allowed in the 10th and 20th minutes, which will not be counted in the total duration of the session. During these pauses, the therapists will monitor cardiovascular parameters, effort perception, and pain. However, HR will be monitored throughout the session so as not to exceed the maximum submaximal values (75% of maximum frequency) and so that training can occur by maintaining target frequency of 50% of maximum HR [14].

Before training (in rest), individuals will be assessed regarding cardiovascular parameters and will be reassessed in the 1st and 5th minutes after the end of the session by the study therapists. The control group will be submitted to training by treadmill during a 30-min session without inclination three times per week for 6 weeks.

Experimental group 1 - treadmill gait training with inclination of 5%

Participants in experimental group 1 will perform the same gait training described for the control group; however, the treadmill will have an anterior inclination equivalent to 5% [27]. The treadmill will be inclined gradually in the initial minutes of each session, and the speed will also be adjusted in accordance with the previously described protocol. Experimental group 1 will be submitted to treadmill training during 30-min sessions with an inclination of 5% three times per week for 6 weeks.

Experimental group 2 - treadmill gait training with inclination of 10%

Participants in experimental group 2 will perform the same gait training described for the control group; however, the treadmill will have an anterior inclination equivalent to 10% [28]. The treadmill will be inclined gradually in the initial minutes of each session, and the speed will also be adjusted in accordance with the previously described protocol. Experimental group 2 will be submitted to treadmill training during 30-min sessions with an inclination of 10% three times per week for 6 weeks.

**Fig. 3:**
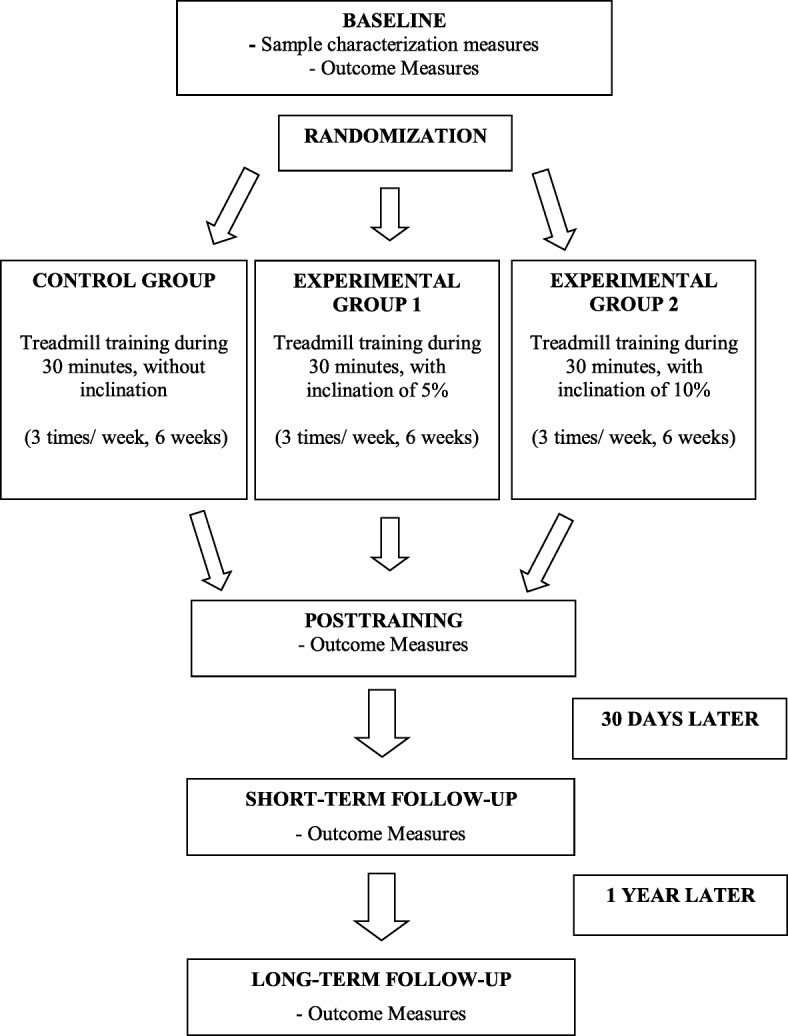
The schematic study design

All participants (of the three groups) will be instructed not to perform any other type of aerobic activity or gait training during the study period. All participants will be called by phone to confirm each training session and all of the evaluations (especially the follow-up). The schematic study design is shown in Fig. 3.

## Statistical analysis

Data analysis will be carried out by a blinded evaluator regarding group allocation by using the Statistical Package for the Social Sciences software (SPSS, IBM, Armonk, NY, USA) for Windows. Only the main study researcher (not involved in data collection) can make the final decision to stop the trial and will have access to the final data.

Descriptive analysis of the sample characterization variables will be performed through central tendency and dispersion measures. Inferential analysis will consider statistical significance of 5% for all studied variables. Mixed analysis of variance (ANOVA) with repeated measures will be used to compare values and variations of outcome measures, comparing values between groups and between baseline, post-training, and follow-up assessments. Normality tests (Shapiro–Wilk) will be used for outcomes and will be compared between groups within each training session by using intergroup comparisons, *t* tests for independent samples, or Mann–Whitney tests.

Group description will be presented as mean and standard deviation, and the effect size and the 95% confidence interval will be reported. Intention-to-treat analysis will be performed for dropout data, considering the last data obtained from the participant.

## Discussion

The proposed study should benefit participants not only in physical aspects but also in psychological and social aspects. It is believed that treadmill training, and especially inclined treadmill training, may result in an improvement in aerobic capacity in patients with stroke and in a reduction in HR and systemic arterial blood pressure values. It is also expected that there will be an improvement in functional parameters, including increased gait speed and functional capacity, promoting ambulation with lower energy expenditure, improving performance in daily living activities, and thus reducing functional dependence of these individuals. Finally, an improvement in quadriceps muscle strength can be expected as well as improvement in quality of life as a reflection of the improvements in the described parameters.

For the scientific community and rehabilitation professionals, it is expected to provide scientific evidence as a proposal to optimize physiotherapeutic practice, presenting a viable, safe, and effective resource for use in the recovery of patients with chronic stroke.

The impossibility of using gas exchange analysis as a gold standard measure of cardiorespiratory fitness can be indicated as the major study limitation. The use of other fitness measures (HR, BP, and exercise capacity) takes into account the clinical applicability of this research given that the gas exchange analysis equipment is costly and so is not feasible to be used in clinical practice. The proposed training time (6 weeks) can be considered a potential (minor) study limitation. Six weeks is considered the minimum length of time suggested for the development of cardiovascular changes, but training duration may be slightly longer for untrained subjects [54]. However, given that is proposed a training on an inclined treadmill (i.e., at a high intensity), it is intended to identify whether 6-week training is quite enough to promote beneficial changes in patients with chronic stroke.

It is important to highlight that in case any negative effects occur, participants who suffer harm from trial participation will receive physical assistance according to the injury. Data results and conclusions will be communicated to participants by email and by journal publications, including for health-care professionals, researchers, and the public in general.

## Trial status

Data collection started shortly after the protocol’s ethical approval starting in August 2017 and is scheduled to end in August 2019.

## Additional file

Additional file 1: Standard Protocol Items: Recommendations for Interventional Trials (SPIRIT) 2013 Checklist: Recommended items to address in a clinical trial protocol and related documents*. (DOCX 52 kb)

## Abbreviations

BP: Blood pressure
FAC: Functional Ambulation Category
HR: Heart rate
HRsub: Submaximal heart rate
SPIRIT: Standard Protocol Items: Recommendations for Interventional Trials
